# Development, characterization, and transferability of SSR markers for *Vriesea carinata* (Bromeliaceae) based on RNA sequencing

**DOI:** 10.1002/aps3.1184

**Published:** 2018-10-16

**Authors:** Cristina C. Todeschini, José L. B. Parizotto, Frank Guzman, Camila M. Zanella, Rogério Margis, Márcia Goetze, Gecele M. Paggi, Laís M. Santana Costa, Camila de Aguiar Melo, Luiza D. Hirsch, Fernanda Bered

**Affiliations:** ^1^ Programa de Pós‐graduação em Genética e Biologia Molecular, Instituto de Biociências Universidade Federal do Rio Grande do Sul Avenida Bento Gonçalves 9500, P.O. Box 15053 91501‐970 Porto Alegre Rio Grande do Sul Brazil; ^2^ Programa de Pós‐graduação em Biologia Celular e Molecular, Centro de Biotecnologia Universidade Federal do Rio Grande do Sul Porto Alegre Rio Grande do Sul Brazil; ^3^ National Institute of Agricultural Botany (NIAB) Huntingdon Road CB3 0LE Cambridge United Kingdom; ^4^ Departamento de Biofisica Universidade Federal do Rio Grande do Sul Porto Alegre Rio Grande do Sul Brazil; ^5^ Ciências Biológicas, Campus do Pantanal, Universidade Federal de Mato Grosso do Sul CP 252 79304‐902 Corumbá Mato Grosso do Sul Brazil

**Keywords:** *Aechmea*, *Alcantarea*, *Bromelia*, Bromeliaceae, *Dyckia*, microsatellite markers, *Neoregelia*, RNA‐Seq, *Vriesea carinata*

## Abstract

**Premise of the Study:**

Expressed sequence tag–simple sequence repeat (EST‐SSR) markers were isolated for *Vriesea carinata*, an endemic bromeliad from the Brazilian Atlantic Forest. These SSR loci may be used to investigate the genetic diversity and population structure of this species and related bromeliads.

**Methods and Results:**

Based on the transcriptome data of *V. carinata*, 30 primer pairs were designed and selected for initial validation. Of these primer pairs, 16 generated suitable SSR loci in 69 individuals. The number of alleles per locus ranged from one to 13; the levels of observed and expected heterozygosity per locus ranged from 0.000 to 1.000 and from 0.000 to 0.935, respectively. All loci produced heterologous amplification. Transferability of the loci was tested in 15 species belonging to three Bromeliaceae subfamilies.

**Conclusions:**

The developed EST‐SSR markers revealed polymorphism in the four studied populations and could be useful to investigate the genetic diversity of *V. carinata* and related species. The markers may also be suitable for novel gene annotation and discovery.

Bromeliaceae is a Neotropical angiosperm family comprising approximately 58 genera and 3352 species distributed in eight subfamilies (Luther, 2012). *Vriesea* Lindl. is part of the monophyletic Tillandsioideae subfamily and is the third largest genus endemic to Brazil, with the Atlantic Forest being its primary center of diversity (Barfuss et al., 2016). *Vriesea carinata* Wawra is an epiphyte or terrestrial species that exists in mesophilic environments and well‐preserved habitats with high humidity distributed along the Atlantic Forest. This species is pollinated by hummingbirds and often used as an ornamental plant, which causes them to be illegally extracted. The increased extractivism of this species, linked to the fragmentation of the Atlantic Forest, has caused concern with respect to the conservation of *V. carinata*, as well as many other bromeliad species (Zanella et al., 2012).

To date, slightly more than a dozen studies have characterized simple sequence repeat (SSR) loci in Bromeliaceae. Of these studies, only three have investigated the subfamily Tillandsioideae (Boneh et al., 2003; Palma‐Silva et al., 2007; Neri et al., 2015), and no loci have been tested for cross‐amplification in *V. carinata*. Considering the high rate of transferability of SSR loci within the same subfamily in Bromeliaceae (Zanella et al., 2012; Goetze et al., 2013; Neri et al., 2015), the development of SSRs in Tillandsioideae may provide markers that can be used in a range of species and that will be valuable tools in the study of species delineation, phylogeography, variation in mating systems, and the detection of hybridization and introgression (Zanella et al., 2012). In this report, we describe the development of 16 expressed sequence tag (EST)–SSR markers for *V. carinata* using next‐generation transcriptome sequencing. Additionally, we tested the transferability of these markers to other bromeliad species.

## METHODS AND RESULTS

Total RNA was isolated from *V. carinata* leaves as described by Guzman et al. (2013). RNA quality was then assessed by 1% agarose gel electrophoresis and quantified by the Thermo Scientific NanoDrop Lite Spectrophotometer (NanoDrop Technologies, Wilmington, Delaware, USA). Subsequently, an RNA‐Seq library was sequenced using the Illumina HiSeq 2000 platform (Illumina, San Diego, California, USA) by Fasteris (Geneva, Switzerland) and assembled de novo in transcripts using the CLC Genome Workbench version 4.0.2 (QIAGEN, Hilden, Germany) as reported by Guzman et al. (2013). All sequence information was deposited in the National Center for Biotechnology Information (NCBI) Sequence Read Archive (SRA; PRJNA167588). From this library, a preliminary analysis of the presence of microsatellites and the determination of the functions of each fragment was undertaken using the Blast2GO tool (Conesa et al., 2005), which generated a draft of potential sequences for the isolation of microsatellites. Preliminary sequences were analyzed, and the primers encompassing the microsatellite regions were designed using Primer3 software (Koressaar and Remm, 2007). Forward primers were synthesized with a 19‐bp M13 tail (5′‐CACGACGTTGTAAAACGAC‐3′) at the 5′ end to enable labeling with a tailed fluorescent dye (FAM, NED, VIC, or PET) during amplification and multiplex genotyping procedures.

To test the isolated SSR loci, fresh leaves were collected from 69 individuals from four natural populations of *V. carinata* (Appendix 1) and stored in silica gel. Total genomic DNA was extracted according to Doyle and Doyle (1990). The PCR reactions were performed in a final volume of 10 μL, containing 10 ng of genomic DNA, 1× enzyme buffer, 2 mM MgCl_2_, 0.2 mM dNTPs, 10 μM of each primer (forward and reverse), and 0.5 unit of *Taq* polymerase (GoTaq; Promega Corporation, Madison, Wisconsin, USA). The amplification protocol was performed according to Palma‐Silva et al. (2007), with the exception of loci Vcar_31 and Vcar_36. The PCR reaction for these loci was performed with an initial denaturation at 95°C for 3 min; 35 cycles of denaturation at 94°C for 30 s, annealing at 60°C for 30 s, and extension at 72°C for 30 s; and a final extension of 72°C for 10 min. PCR was repeated for loci with positive amplification by adding the universal M13 primer labeled with different fluorochromes (FAM, VIC, PET, or NED) according to Goetze et al. (2013). The amplification products were genotyped in an ABI 3500 DNA Genetic Analyzer (Applied Biosystems, Foster City, California, USA) and compared to GeneScan 500 LIZ Size Standard, with allele identification performed in GeneMarker software Demo version 1.97 (SoftGenetics, State College, Pennsylvania, USA). To describe the variation of the microsatellites, observed and expected heterozygosities were estimated for each locus and population using the software MSA (Dieringer and Schlötterer, 2003). FSTAT (Goudet, 1995) was used to estimate the number of alleles, and GENEPOP ON THE WEB (Raymond and Rousset, 1995) was used to test for Hardy–Weinberg equilibrium and linkage disequilibrium and to estimate the inbreeding coefficient (*F*_IS_).

Based on the transcriptome data of *V. carinata*, 30 primer pairs were designed and tested, of which 16 yielded suitable SSR loci in 69 individuals among the four studied populations (Tables 1, 2). The number of alleles encountered ranged from one to 13 in the four populations evaluated (Table 2). Levels of observed and expected heterozygosity varied from 0.000 to 1.000 and from 0.000 to 0.935, respectively (Table 2). Four loci showed significant deviation of Hardy–Weinberg equilibrium in at least one of the analyzed populations (*P* < 0.001) (Table 2). Significant linkage disequilibrium was detected only between Vcar_31 and Vcar_93 and between Vcar_91 and Vcar_293. Linkage disequilibrium could not be tested by Fisher's test in Vcar_501 and Vcar_395 and in three pairs of loci with Vcar_139 (Vcar_31, Vcar_93, and Vcar_258). The 16 SSRs were tested in other bromeliads in order to verify their potential for heterologous amplification. Of the 16 characterized loci, seven amplified in more than 50% of the species (Table 3). The locus Vcar_36 amplified in all species followed by Vcar_501 (14), Vcar_153 (13), Vcar_258 (9), and Vcar_115/Vcar_143/Vcar_395, with each amplifying in eight species. All 16 loci amplified in *V. reitzii* Leme & A. F. Costa, and approximately 81% amplified in all five individuals tested (Table 4). These loci also amplified in some species belonging to subfamilies other than Tillandsioideae, which suggests their potential utility in genetic studies of populations involving other bromeliad subfamilies.

**Table 1:** Characteristics of the 16 microsatellite markers developed for *Vriesea carinata*

Locus^a^	Primer sequences (5′–3′)	Repeat motif	Allele size range (bp)	Putative function [Organism]	*E*‐value	GenBank accession no.
Vcar_10	F: AACTTGTCTATGTCTAAAGGAATGG	(TTG)_5_N(GTT)_9_N(TAT)_7_	243–261	Endoribonuclease Dicer homolog 1 isoform X1 [*Ananas comosus*]	0.0	MF563945
	R: ACTCTGCGGCTGTTCTTCTC					
Vcar_31	F: CCGTAGGCGACGATAGAGAG	(AG)_15_	206–236	E3 SUMO‐protein ligase SIZ1 [*Ananas comosus*]	0.0	MF563947
	R: GTGGGGGAGGAGAGAGAAAG					
Vcar_36	F: CCGAAGTCTCTCCCTTTTCC	(CGC)_7_	262–265	Flowering time control protein FPA [*Ananas comosus*]	0.0	MH319796
	R: TCATTCGACTGCTTCTGCTG					
Vcar_72	F: TTCTCAATCTTCACCGACGA	(CGC)_7_N(GTCCTC)_3_N(TCC)_6_	228–237	Histone acetyltransferase GCN5 [*Ananas comosus*]	0.0	MF563948
	R: GCGAGGAGGACGATGACTC					
Vcar_91	F: CGACCCACTCTTCGCTAGTC	(ACG)_10_	186–213	Adagio‐like protein 3 [*Ananas comosus*]	0.0	MF563949
	R: ATGCGGGAGTCCTCCTTACT					
Vcar_93	F: TTGCCACAAAGACGTACCAA	(AG)_8_	229–259	Coronatine‐insensitive protein homolog 1b‐like [*Ananas comosus*]	0.0	MF563950
	R: TGCTGGGAAGAGGTCAGACT					
Vcar_95.1	F: CATTGGTGTTGTTGGGTTCA	(TGCCAC)_4_N(CTT)_6_	200–224	Calmodulin‐like [*Gossypium raimondii*]	1e‐97	MF563951
	R: GAGATGGCTGAGGAAGATGC					
Vcar_115	F: CGCCATTATCAACGATCACA	(GA)_8_N(AG)_7_	200–222	Leucine‐rich repeat receptor‐like serine/threonine/tyrosine‐protein kinase [*Ananas comosus*]	6e‐171	MF563952
	R: GGCGCTTATTTATGCTCTGC					
Vcar_139	F: CCCCGAAATTGATCGAAAC	(AGG)_6_N(TGG)_9_N(GA)_8_	246–264	Homogentisate phytyltransferase 2, chloroplastic isoform X1 [*Ananas comosus*]	0.0	MF563953
	R: GGTGAGTGAGATTGGGTGGT					
Vcar_143	F: CCCTTCCCGATCGAATTTAT	(CTC)_9_N(TAGGGT)_2_	240–264	Probable serine/threonine‐protein kinase WNK4 [*Elaeis guineensis*]	4e‐86	MF563954
	R: AGGAGCTCCGATCCATAACC					
Vcar_153	F: TGGATGTGAAGGAGCAGAAA	(GCTGAA)_2_N(GCT)_8_N(CCTGCT)_2_	222–234	Copper transport protein ATX1 [*Phalaenopsis equestris*]	7e‐18	MF563955
	R: ATGCAACAATCATGCAGTGG					
Vcar_258	F: TTCTTCGAGAGCTTCGATCC	(CCT)_9_	228–237	Monothiol glutaredoxin‐S14, chloroplastic [*Ananas comosus*]	4e‐96	MF563956
	R: GAGTCAATGCGGAGAAGCAT					
Vcar_280.1	F: CACAAAGCCACTCACAAAACC	(CT)_8_N(TC)_7_	220–248	Basic blue protein‐like isoform X1 [*Ananas comosus*]	4e‐78	MF563957
	R: ACTGCCAATCCGGTATGAAG					
Vcar_293	F: TTCGGGAGCTCTAGGGTTTT	(CCT)_9_	244–260	DNA mismatch repair protein PMS1 isoform X3 [*Ananas comosus*]	2e‐142	MH319797
	R: CACCAACTCCTTCACAGCAG					
Vcar_395	F: CGTATCTCCTCGTCGCTCTC	(TTC)_12_	224	Transcription factor HY5‐like [*Ananas comosus*]	2e‐91	MH319798
	R: CTGATCAGCTCCCCTTCC					
Vcar_501	F: GATTCATGCGCGAGATTGTA	(AGAA)_6_	130	MADS‐box protein JOINTLESS‐like [*Ananas comosus*]	3e‐30	MH319799
	R: ACGGTGGTAGCTCCAATACG					

a All loci except Vcar_31 and Vcar_36 were amplified using a touchdown program with a temperature range between 58°C and 48°C (Palma‐Silva et al., 2007).

**Table 2:** Characterization of the 16 microsatellite loci in four populations of *Vriesea carinata*.^a^

Loci	Morretes (*n* = 21)				Santa Virgínia (*n* = 24)				Bertioga (*n* = 11)				Ubatuba (*n* = 13)			
	*A*	*H*_o_	*H*_e_	*F*_IS_	*A*	*H*_o_	*H*_e_	*F*_IS_	*A*	*H*_o_	*H*_e_	*F*_IS_	*A*	*H*_o_	*H*_e_	*F*_IS_
Vcar_10	7	0.762	0.811	0.062	2	0.000	0.667	1.000	7	0.636	0.792	0.204	7	1.000	0.837	−0.210
Vcar_31	9	0.444	0.869	0.504^b^	13	0.667	0.927	0.287	7	0.500	0.837	0.416	4	0.200	0.778	0.765
Vcar_36	2	0.190	0.176	−0.081	2	0.043	0.043	0	NT	NT	NT	NT	NT	NT	NT	NT
Vcar_72	4	0.263	0.633	0.591^b^	4	0.125	0.642	0.816^b^	3	0.300	0.652	0.554	4	0.363	0.636	0.441
Vcar_91	5	0.176	0.451	0.616	2	0.062	0.062	0	1	0.000	0.000	—	3	0.600	0.611	0.018
Vcar_93	12	0.809	0.883	0.085	12	0.545	0.935	0.429	11	0.909	0.909	0.000	11	0.833	0.913	0.091
Vcar_95.1	7	0.667	0.756	0.121	3	1.000	0.733	−0.500	5	0.454	0.679	0.342	5	0.727	0.749	0.030
Vcar_115	7	0.550	0.608	0.097	5	0.353	0.684	0.492	3	0.364	0.554	0.355	8	0.444	0.745	0.418
Vcar_139	5	0.375	0.842	0.571^b^	1	0.000	0.000	—	4	0.000	0.800	1.000	5	0.500	0.768	0.359
Vcar_143	6	0.428	0.449	0.048	—	—	—	—	3	0.400	0.542	0.273	6	0.833	0.717	−0.170
Vcar_153	5	0.333	0.561	0.412	3	0.350	0.509	0.318	4	0.454	0.762	0.415	2	0.308	0.443	0.314
Vcar_258	3	0.571	0.648	0.122	2	0.000	0.667	1.000	3	0.428	0.659	0.368	3	0.500	0.750	0.368
Vcar_280.1	9	0.067	0.811	0.920^b^	3	0.000	0.800	1.000	4	0.000	0.747	1.000^b^	9	0.500	0.908	0.467
Vcar_293	3	0.450	0.619	0.278	5	0.500	0.833	0.423	NT	NT	NT	NT	NT	NT	NT	NT
Vcar_395	1	0.000	0.000	—	1	0.000	0.000	—	NT	NT	NT	NT	NT	NT	NT	NT
Vcar_501	1	0.000	0.000	—	—	—	—	—	NT	NT	NT	NT	NT	NT	NT	NT
Mean	5.375	0.380	0.570	0.310	4.143	0.260	0.536	0.526	4.583	0.370	0.661	0.448	5.583	0.567	0.738	0.241

*Note:*

— = index could not be calculated; *A* = number of alleles; *F*_IS_ = inbreeding coefficient; *H*_e_ = expected heterozygosity; *H*_o_ = observed heterozygosity; *N* = number of plants sampled; NT = locus not tested for the population (due to problems with the DNA of some individuals, the last isolated loci could not be tested in these populations).

a Locality and voucher information are provided in Appendix 1.

b Inbreeding coefficient (*F*_IS_) departed significantly from Hardy–Weinberg equilibrium (*P* < 0.001).

**Table 3:** Heterologous amplification of the 16 isolated loci of *Vriesea carinata* in 15 species of the subfamilies Bromelioideae, Pitcairnioideae, and Tillandsioideae of Bromeliaceae.^a^

Locus	Bromelioideae						Pitcairnioideae					Tillandsioideae				Total
	*Aechmea calyculata*	*Aechmea caudata*	*Aechmea comata*	*Aechmea kertesziae*	*Bromelia antiacantha*	*Neoregelia laevis*	*Dyckia divaricata*	*Dyckia excelsa*	*Dyckia grandidentata*	*Dyckia leptostachya*	*Dyckia pottiorum*	*Alcantarea extensa*	*Vriesea altodaserrae*	*Vriesea philippocoburgii*	*Vriesea reitzii* b	
Vcar_10	—	w	—	—	—	—	—	—	—	—	w	—	—	—	w/+	3
Vcar_31	—	+	—	—	—	—	—	—	—	—	+	+	—	—	+	4
Vcar_36	+	+	+	+	+	+	w	w	+	+	w	w	+	+	+	15
Vcar_72	—	—	—	—	—	—	—	—	—	—	—	—	—	—	+	1
Vcar_91	—	—	—	w	—	—	w	+	—	w	+	—	—	—	w/+	6
Vcar_93	—	—	—	—	—	w	—	—	—	w	—	—	w	w	—/w	5
Vcar_95.1	—	—	—	—	—	—	—	—	—	—	—	—	+	+	+	3
Vcar_115	w	—	—	—	—	w	—	w	—	—	w	+	+	+	+	8
Vcar_139	—	w	—	—	w	—	—	—	—	—	—	w	—	—	w/+	4
Vcar_143	—	w	w	—	—	—	—	w	—	+	—	+	+	+	w/+	8
Vcar_153	+	w	—	—	+	+	+	+	w	+	+	+	+	+	+	13
Vcar_258	—	w	w	—	+	—	w	+	—	+	w	+	—	—	w/+	9
Vcar_280.1	—	—	—	—	—	—	—	—	—	—	—	—	—	—	+	1
Vcar_293	—	—	—	w	—	—	—	—	—	—	w	—	—	—	+	3
Vcar_395	w	w	—	w	+	—	+	—	w	+	—	—	—	—	w/+/—	8
Vcar_501	+	+	+	+	+	+	+	+	—	+	+	+	+	+	w/+/—	14

*Note:*

+ = successful amplification; — = unsuccessful amplification; w = weak amplification.

a Locality and voucher information are provided in Appendix 1.

b Because five individuals were tested in *Vriesea reitzii*, there is variation in the results (see Table 4).

**Table 4:** Heterologous amplification of the 16 isolated loci of *Vriesea carinata* in five individuals of *V. reitzii*

Loci	*Vriesea reitzii* 1	*Vriesea reitzii* 2	*Vriesea reitzii* 3	*Vriesea reitzii* 4	*Vriesea reitzii* 5	Range in *V. reitzii* (bp)^a^
Vcar_10	w	w	w	+	+	250–274
Vcar_31	+	+	+	+	+	220–230
Vcar_36	+	+	+	+	+	262–265
Vcar_72	+	+	+	+	+	228–251
Vcar_91	w	w	+	+	w	240–249
Vcar_93	—	w	w	w	w	246–251
Vcar_95.1	+	+	+	+	+	239–250
Vcar_115	+	+	+	+	+	200–220
Vcar_139	+	+	w	+	+	252–255
Vcar_143	w	+	+	+	+	249–255
Vcar_153	+	+	+	+	+	234–240
Vcar_258	w	+	w	+	+	231–237
Vcar_280.1	+	+	+	+	+	220–226
Vcar_293	+	+	+	+	+	256–260
Vcar_395	w	—	—	+	+	350
Vcar_501	+	+	w	—	w	350–400

*Note:*

+ = successful amplification; — = unsuccessful amplification; w = weak amplification.

a Respective size ranges. Cross‐amplification tests were performed using 2% agarose gel and a 50‐bp ladder.

All markers were tested for their transferability in one individual of 15 species belonging to three Bromeliaceae subfamilies. The conditions of the PCR amplifications were the same as described above. The amplification products were run on 2.0% agarose gel electrophoresis, stained with GelRed (Biotium, Hayward, California, USA), and compared to 100‐bp and 50‐bp ladders (Ludwig Biotechnology Ltda., Alvorada, Rio Grande do Sul, Brazil). The loci were considered to have positive amplification when at least one band of the expected size was visualized. Additionally, cross‐amplification was tested in five individuals of *V. reitzii* to assess potential individual variation. Voucher information concerning the species investigated is listed in Appendix 1.

## CONCLUSIONS

The 16 SSR markers described in this study revealed polymorphism in the studied populations of *V. carinata* and can be useful for the study of genetic diversity and evolution in other related species. Additionally, the loci described in this study may be used in studies that promote conservation and management of *V. carinata*, which is increasingly threatened by extractivism and habitat destruction. Moreover, due to association with coding sequences, the EST‐SSRs isolated in this study have the potential for direct gene tagging and can facilitate future functional genomic studies in *V. carinata*.

## DATA ACCESSIBILITY

All sequence information was uploaded to the National Center for Biotechnology Information (NCBI) Sequence Read Archive (accession no. PRJNA167588); primer sequences were uploaded to GenBank, and accession numbers are provided in Table 1.

## APPENDIX 1 Locality and voucher information for the species used in this study.

**Table:**

Species	Locality	*N*	Geographic coordinates	Voucher no.^a^
*Aechmea calyculata* (E. Morren) Baker	Eight Waterfalls Park, São Francisco de Paula, RS, Brazil	1	29°26′S, 50°33′W	ICN 165253
*Aechmea caudata* Lindm.	Florianópolis, SC, Brazil	1	27°26′S, 48°24′W	ICN 187561
*Aechmea comata* (Gaudich.) Baker	Florianópolis, SC, Brazil	1	26°26′S, 48°31′W	ICN 184897
*Aechmea kertesziae* Reitz	Laguna, SC, Brazil	1	28°30′S, 48°45′W	ICN 167498
*Alcantarea extensa* (L. B. Sm.) J. R. Grant	Biological Station of Santa Lúcia, Santa Tereza, ES, Brazil	1	40°53′S, 19°97′W	MBML 25417
*Bromelia antiacantha* Bertol.	Mafra, SC, Brazil	1	26°06′S, 59°19′W	HBR 4067
*Neoregelia laevis* (Mez) L. B. Sm.	Graciosa highway, Morretes, PR, Brazil	1	25°47′S, 48°83′W	ICN 190907
*Dyckia divaricata* Leme & Büneker	Antônio João, MS, Brazil	1	22°07′S, 56°05′W	ICN 187138
*Dyckia excelsa* Leme	Bodoquena, MS, Brazil	1	20°47′S, 56°37′W	ICN 187137
*Dyckia grandidentata* P. J. Braun & Esteves	São Gabriel do Oeste, MS, Brazil	1	19°18′S, 54°48′W	ICN 187129
*Dyckia leptostachya* Baker	Porto Alegre, RS, Brazil	1	30°07′S, 51°14′W	ICN 187141
*Dyckia pottiorum* Leme	Corguinho, MS, Brazil	1	19°43′S, 54°54′W	ICN 187134
*Vriesea altodaserrae* L. B. Sm.	Rio Manso, Joinville, SC, Brazil	1	26°28′S, 49°14′W	FURB01147
*Vriesea carinata* Wawra	Bertioga, SP, Brazil	11	23°51′S, 46°08′W	ICN 177670
	Graciosa Mountain, Morretes, PR, Brazil	21	25°28′S, 48°50′W	ICN 177669
	Ubatuba, SP, Brazil	13	23°26′S, 45°50′W	RB00265965
	Parque Estadual da Serra do Mar, Núcleo Santa Virgínia, São Luiz do Paraitinga, SP, Brazil	24	23°20′S, 45°09′W	ESA083165
*Vriesea philippocoburgii* Wawra	Morretes, PR, Brazil	1	25°28′S, 48°50′W	MBM33325
*Vriesea reitzii* Leme & Costa	São Francisco de Paula, RS, Brazil	5	29°44′S, 50°58′W	ICN 190912

ES = Espírito Santo; MS = Mato Grosso do Sul; *N* = number of individuals sampled; PR = Paraná; RS = Rio Grande do Sul; SC = Santa Catarina; SP = São Paulo.

a Herbarium acronyms are per Index Herbariorum (Thiers, 2018).
